# A PILOT STUDY TO TEST METHODS FOR DATA COLLECTION ON NURSES CARING FOR RESIDENTS WITH DEMENTIA IN THE NURSING HOME

**DOI:** 10.1093/geroni/igz038.1691

**Published:** 2019-11-08

**Authors:** Rebekah Perkins, Elizabeth Cashdan, Katherine Supiano

**Affiliations:** University of Utah, Salt Lake City, Utah, United States

## Abstract

Nurses draw from their experiences and intuition to detect changes in patient condition, patterns of patient behaviors, and evidence of distress. In the nursing home setting, nurses care for residents with dementia and manage challenging behavioral and psychological symptoms of dementia (BPSD), and may rely on informed intuition to assess and capably respond to such behaviors. To date, no observational method has been developed to discern nurse-resident interactions that identify expert nurses who effectively address BPSD. It is not known if or how nurses in this setting use intuition to make clinical judgments and decisions to manage BPSD events. Using an ethogram approach, we developed an observational tool and spot interview method to discern BPSD events, background and proximal factors and nurse responses to BPSD. Pilot observations took place over three nursing shifts to identify nurse-resident interactions during BPSD events using the observation tool. Nurse-resident interactions were followed by spot interviews with each nurse to clarify their responses to BPSD. Semi-structured interviews were conducted with nurse participants to further develop an interview guide and identify elements of nurse intuition. The pilot study affirmed the feasibility of gaining access to facilities with residents with BPSD, of nurse comfort with field observation and interviews, and established preliminary construct validity of the “expert nurse.” Verification of the utility of this observation and interview method permit further examination of effective nurse engagement with nursing home residents with BPSD, informs our understanding of nurse intuition and permits further exploration of the broader context of BPSD.

